# Intrinsic Delocalization during the Decay of Excitons in Polymeric Solar Cells

**DOI:** 10.3390/polym8120414

**Published:** 2016-11-30

**Authors:** Weikang Chen, Deyao Jiang, Renai Chen, Sheng Li, Thomas F. George

**Affiliations:** 1 Department of Physics, Zhejiang Normal University, Jinhua 321004, Zhejiang, China; cwk90wcl@163.com (W.C.); jdy1210@163.com (D.J.); lovuitchen@gmail.com (R.C.); 2 Department of Physics, Fudan University, Shanghai 200433, China; 3 Office of the Chancellor and Center for Nanoscience, Departments of Chemistry & Biochemistry and Physics & Astronomy, University of Missouri–St. Louis, St. Louis, MO 63121, USA

**Keywords:** delocalization, excitons, polymeric solar cells, dipole moment

## Abstract

In bulk heterojunction polymer solar cells, external photoexcitation results in localized excitons in the polymer chain. After hot exciton formation and subsequent relaxation, the dipole moment drives the electron to partially transfer to extended orbitals from the original localized ones, leading to self-delocalization. Based on the dynamic fluorescence spectra, the delocalization of excitons is revealed to be an intrinsic property dominated by exciton decay, acting as a bridge for the exciton to diffuse in the polymeric solar cell. The modification of the dipole moment enhances the efficiency of polymer solar cells.

## 1. Introduction

For two decades, scientists have worked on improving the efficiency of organic solar cells [1,2,3,4,5,6,7,8,9], where, in particular, there has been great progress in the application of semiconducting conjugated polymers in this field [4,5,8,10]. For the first generation of organic solar cells, the power conversion efficiencies were lower than 0.7% [11,12], where their simple structure resembles a sandwich, in that only a single organic layer is inserted between two metal electrodes [2,3].

Once the photoactive layer undergoes photoexcitation, aside from charge generation and singlet fission in organic solar cells, it mostly results in an electron–hole pair or an exciton. After exciton diffusion, the fabricated heterojunction dissociates the exciton, and then the resultant charge carriers freely move towards the cathode and anode.

**Figure 1:**
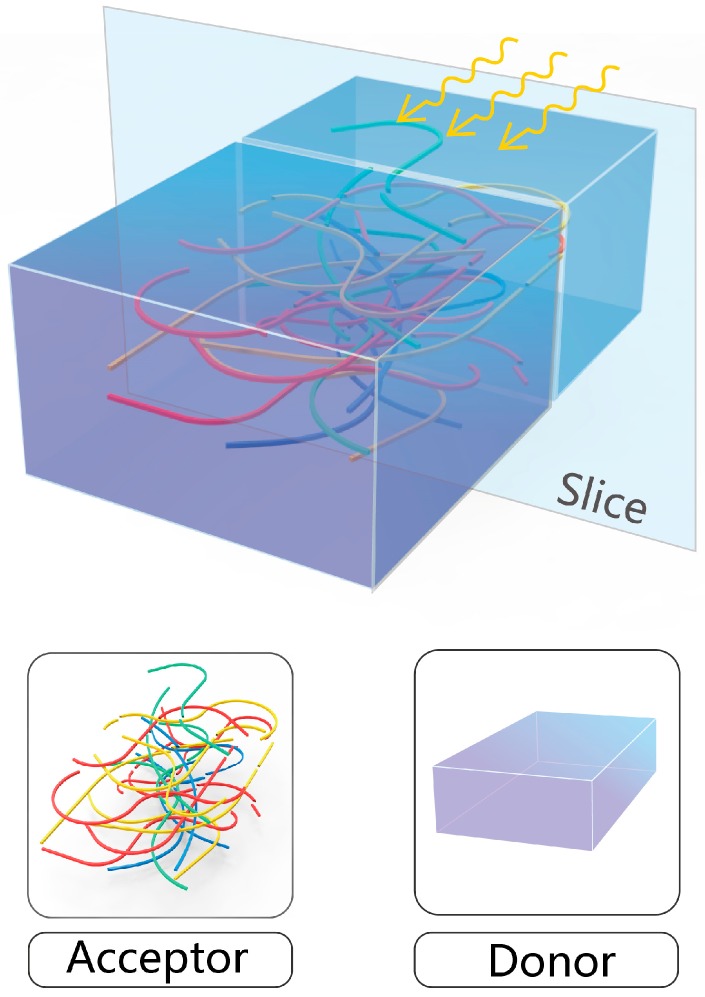
Diagrammatic layout of a bulk heterojunction solar cell.

During the exciton diffusion process, the spontaneous transition or nonradiative transition leads to the exciton’s decay. So, reducing the length of exciton diffusion is desirable to cut down on exciton decay, and then to improve the efficiency of the organic solar cell. Given that, a bulk heterojunction solar cell has been proposed whose layout is sketched in Figure 1, where the conjugated polymers—one-dimensional-morphological acceptors—are “disorderedly” dissolved in the donor domain to form a realistic bulk heterojunction solar cell. On the basis of this structure, the mechanism regarding exciton diffusion can be briefly depicted: once an exciton or electron–hole pair is formed in an organic solar cell due to photoexcitation, the heterojunctions randomly distributed all over the bulk of solar cells largely reduce the exciton’s diffusion length, making it easy for the exciton to transfer and reach the heterojunction, eventually being separated into charged carriers at the interface.

**Figure 2:**
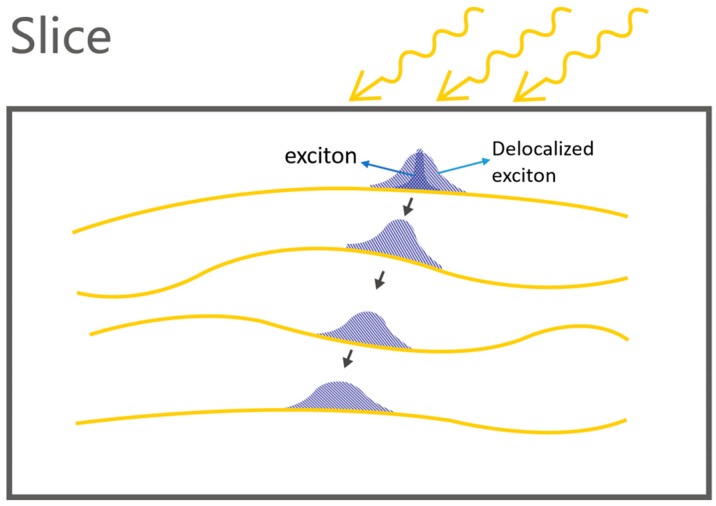
Hypothesis for exciton delocalization and diffusion in a bulk heterojunction solar cell.

Thus, during the whole dynamical process, it becomes crucial to clarify how the exciton is formed in a solar cell and diffuses to the heterojunction of solar cells. One recent experimental study—through femtosecond nonlinear optical spectroscopy and nonadiabatic quantum molecular mechanics—showed that hot excitons are formed within several femtoseconds in the initial excitation, undergoing relaxation on a 10–12 s timescale [13]. The resultant exciton—different from the inorganic semiconductors—is localized in the conjugated polymeric chain by the prominent electron-lattice coupling of conjugated polymers. The dynamical mechanism furthermore reveals that the exciton quickly evolves to a delocalized state [14]. Therefore, a possible physical picture with respect to the exciton diffusion can be assumed in Figure 2, which shows slices cut from the middle of the bulk heterojunction solar cell in Figure 1, where the resultant exciton is first localized along the polymer chain. Following that, the exciton has to be delocalized along the polymer chain. Here, the delocalization of the exciton paves a necessary path for exciton diffusion.

Related experiments have observed some degree of delocalization in the emission pathways of excited states in polymers [15,16]. Especially in 2004, based on the planarity of the polymeric material poly(di(2-ethylhexyloxy)benzo[1,2-b:4,5-b0]dithiophene-cooctylthieno[3,4-c]pyrrole-4,6-dione), i.e., PBDTTPD, exciton delocalization was found to be over ~2 nm [17], which is indeed favorable for exciton diffusion and charge generation in solar cells [17,18]. Most importantly, the latest experiment demonstrated that the charge separation in efficient organic photoconversion systems is largely due to the delocalization of excitons in a single molecule, rather than energy-gradient–driven intermolecular hopping [14]. Gaining an understanding of the delocalization of excitons along the polymer chain is a goal of this article. We want to answer the question as to whether the delocalization of the exciton/excited state is an intrinsic physical property of conjugated polymers.

In 2013, it was shown that the radiative decay of an exciton can efficiently display the intrinsic physical property of a low-dimensional semiconductor, while the extrinsic disturbances hardly contribute to the properties of the exciton [19,20,21,22]. Moreover, various details with respect to the intrinsic physical property—such as decay time—can be extracted from the exciton decay in conjugated polymers [23,24,25,26]. Hence, to link exciton delocalization with exciton decay and the one-dimensional structure of conjugated polymers is the essential task to resolve the questions above.

Our purpose here is to illustrate the exciton decay in a conjugated polymer chain, where the key point is to clarify the assumption that the radiative decay of excitons entails a delocalization process. Based on the developed nonadiabatic fluorescence dynamics combined with electronic transition molecular dynamics, we aim to precisely depict the whole process of exciton radiative decay and its delocalization, as well as its corresponding dynamic fluorescence spectra, and finally to unveil the intrinsic delocalization channel, which acts as bridge for exciton diffusion in solar cells. Clarification of the mechanism of exciton delocalization presents an opportunity for fabrication in nanoscale polymeric solar cells.

## 2. Methodology

We start from the nonadiabatic molecular dynamics and the well-celebrated Su–Schreiffer–Heeger (SSH) Hamiltonian [27] describing the properties of a conjugated polymer:

(1) $$H=-\sum_{l,s}[t_{0}+\alpha (u_{l+1}-u_{l})+{\left(-1\right)}^{l}t_{e}]\times [c_{l+1,s}^{†}c_{l,s}+H.c.]+\frac{K}{2}\sum_{l}{\left(u_{l+1}-u_{l}\right)}^{2}+\frac{M}{2}\sum_{l}{\dot{u}}_{l}^{2}.$$

Here, *t*_0_ is a hopping constant, *α* is an electron-lattice hopping constant, $c_{l+1,s}^{†}\;(c_{l,s})$ denotes the electron creation (annihilation) operator at unit cluster *l* with spin *s*, *u_l_* is the displacement of cluster *l*, *t_e_* is the Brazovskii–Kirova term, *K* is an elastic constant, and *M* is the mass of the *l*th cluster. At each instant for the nonadiabatic calculation, we use the time-dependent Schrodinger equation:

(2) $$i\hbar \frac{\partial }{\partial t}\Psi =H\Psi .$$

The time-dependent electron wavefunction can be written as a linear superposition of $\Phi_{\mu }$,

(3) $$\Psi_{\nu }(t)=\sum_{\mu }c_{\nu \mu }(t)\Phi_{\mu },$$

where $\Phi_{\mu }$ is the eigen-wavefunction of the eigen-equation $H_{e}\Phi_{\mu }=\varepsilon_{\mu }\Phi_{\mu }$ mentioned above. According to the Feynman–Hellmann theorem, the atomic force along the polymer chain is expressed as

(4) $$F_{l}=-\langle \Psi \left|\frac{\partial H}{\partial u_{l}}\right|\Psi \rangle \;.$$

In order to describe the dynamic transition process, we need further to employ the electron transition rate [28] between a high-energy (*E_H_*) and a low-energy (*E_L_*) level,

(5) $$\gamma_{ab}=\frac{4{\left(E_{H}-E_{L}\right)}^{3}}{3\hbar^{4}c^{3}}p^{2}\;,$$

where *p* is the transition dipole moment and *c* is the speed of light. Additionally, the corresponding fluorescence intensity from the radiative transition is

(6) $$F=\Delta E_{w}P_{H}\frac{4{\left(E_{H}-E_{L}\right)}^{3}}{3\hbar^{4}c^{3}}p^{2}\;,$$

where $\Delta E_{w}$ is the energy difference for a specific wavelength between the molecular orbitals |H〉 and |L〉, and $P_{H}$ denotes the electron population occupation of the higher orbital |H〉.

If the two molecular orbitals undergoing the above radiative transition are written as |H〉 and |L〉, then at time *t*—according to nonadiabatic dynamics—orbital |H〉 and |L〉 evolves into |H(t)〉 and |L(t)〉. Shown as the superposition of observer states—that is, of the eigenwavefunctions {|1,0〉,|2,0〉,|3,0〉,…,|n−1,0〉,|n,0〉} in their initial time—the two states are written as

(7) $$|H(t)\rangle =a_{1}(t)|1,0\rangle +a_{2}(t)|2,0\rangle +\cdots a_{n-1}(t)|n-1,0\rangle +a_{n}(t)|n,0\rangle$$

(8) $$|L(t)\rangle =b_{1}(t)|1,0\rangle +b_{2}(t)|2,0\rangle +\cdots b_{n-1}(t)|n-1,0\rangle +b_{n}(t)|n,0\rangle ,$$

where all the zeros in the kets indicate the states at the initial starting time—namely, the observer states.

Thus, to calculate the different components of the fluorescence intensity, we set up a series of eigenstates that remain unchanged for each calculation. Then, the transition dipole moment is rewritten as

(9) $$p=e\langle H\left|r\right|L\rangle =\left(\begin{array}{c} a_{1}^{*}\langle 1,0|+a_{2}^{*}\langle 2,0|+\\ \cdots +a_{n-1}^{*}\langle n-1,0|+a_{n}^{*}\langle n,0| \end{array}\right)\;\left|r\right|\;\left(\begin{array}{c} b_{1}|1,0\rangle +b_{2}|2,0\rangle +\\ \cdots +b_{n-1}|n-1,0\rangle +b_{n}|n,0\rangle \end{array}\right).$$

The specific fluorescence intensity of a certain transition between two different observer states (for example, states |m,0〉 and |n,0〉) can be measured by a spectrograph and expressed as

(10) $$F_{mn}=\Delta E_{w}P_{H}\frac{4{\left(E_{H}-E_{L}\right)}^{3}}{3\hbar^{4}c^{3}}p_{m\to n}^{2},$$

where $p_{m\to n}^{2}=a_{m}^{*}b_{n}^{*}a_{m}b_{n}{\left|\langle m,0|r|n,0\rangle \right|}^{2}$ is the square of the modulus of the sub-dipole moment between the two states, corresponding to $F_{mn}$. Out of this, we can calculate different kinds of wavelength-related dipole moments when excitons undergo radiative transitions, and consequently obtain the corresponding fluorescence intensity. The above coupled equations can fully describe the electron dynamics in the system.

## 3. Results and Discussion

**Figure 3:**
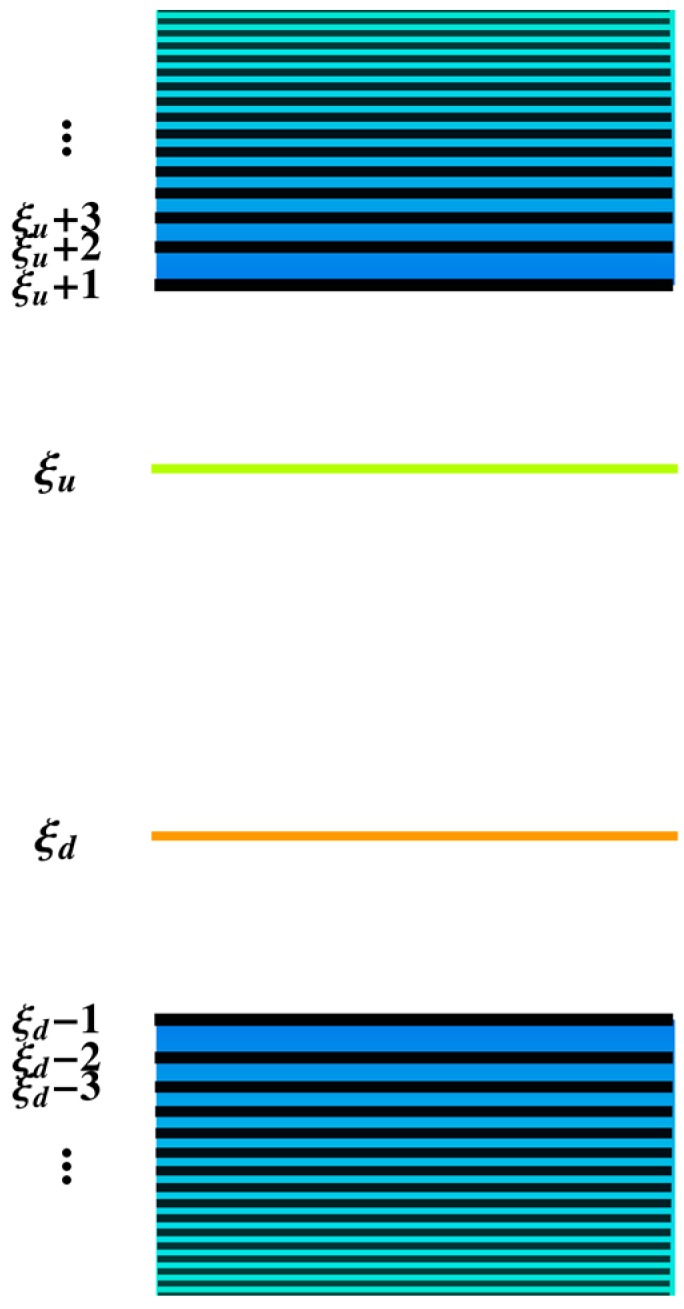
Energy levels of different orbitals when an exciton first forms in a conjugated polymer.

After an external light/laser beam excites a conjugated polymer, the electron in the highest occupied molecular orbital (HOMO) absorbs the energy of an incident photon and transits to the lowest unoccupied molecular orbital (LUMO) [28]. As the excitation is completed, the original HOMO and LUMO states move to the middle of the energy gap to form two localized orbitals of a bound singlet exciton. Figure 3 illustrates the energy levels of the orbitals in the conjugated polymer, where both $\xi_{u}$ and $\xi_{d}$—corresponding to the localized states of the exciton—are depicted in the middle of the gap. During the exciton radiative decay, the fluorescence effect results from many transitions among different orbitals shown in Equation (6), as time evolves, which involves light emission with different wavelengths.

**Figure 4:**
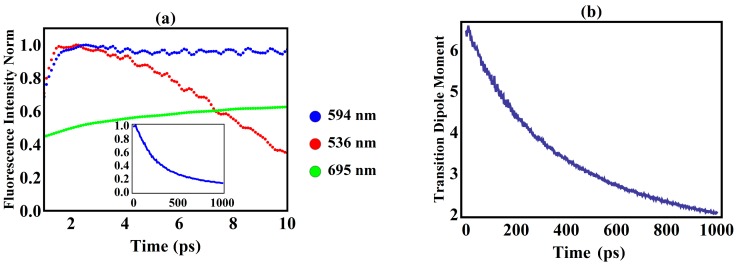
(**a**) Time evolution of the fluorescence intensity for three different wavelengths, where the inset plot is 1 ns evolution for the wavelength of 594 nm; (**b**) Change of the square of the total transition dipole moment during 1 ns (the units of the vertical axis are e^2^Å^2^).

Applying the methods mentioned above, during the singlet exciton decay, the fluorescence spectra of three different wavelengths—594 nm, 536 nm, 695 nm—are obtained within 10 ps, as shown in Figure 4a. Although Figure 4 exhibits the differences of the time-dependent intensities among the three spectra, only the intensity contributed by the spectrum of 594 nm exponentially decays with time, as seen by the inset in Figure 4a, finally decreasing to 0.2 when time reaches 1 ns. This is consistent with recent experimental research [25], whereby the relative lifetime can be seen as ranging from 250 to 500 ps. For the spectrum of 594 nm, Figure 4b shows that the evolution of the transition dipole moment is similar to that of fluorescence over one nanosecond, which provides a trace that the fluorescence is proportional to the magnitude of the total transition dipole moment.

When the radiative transition process is triggered and time evolves, the electron in the higher molecular orbital |H(t)〉 transits to the lower molecular orbital |L(t)〉, emitting a photon. Here it has to be emphasized that during the dynamical process of exciton decay, the molecular orbitals |H(t)〉 and |L(t)〉 are actually each a superposition of all molecular orbitals, as follows:

(11) $$|H(t)\rangle =a_{1}(t)|1,0\rangle +a_{2}(t)|2,0\rangle +\cdots a_{n-1}(t)|n-1,0\rangle +a_{n}(t)|n,0\rangle$$

(12) $$|L(t)\rangle =b_{1}(t)|1,0\rangle +b_{2}(t)|2,0\rangle +\cdots b_{n-1}(t)|n-1,0\rangle +b_{n}(t)|n,0\rangle .$$

This indicates that the electronic transition during the decay of the exciton not only happens between the localized molecular orbitals in the gap, but extends to extensive orbitals outside of the gap that, accordingly, triggers many possible transitions from the higher to lower orbital, such as $|\xi_{u}\rangle$ to $|\xi_{d}\rangle$ and $|\xi_{u}+1\rangle$ to $|\xi_{d}-1\rangle$ in Figure 3. Fortunately, experiments [29,30] have pointed out that the key to understanding the underlying mechanism is the evolution of the transition dipole moment and its relationship with the exciton transition decay.

**Figure 5:**
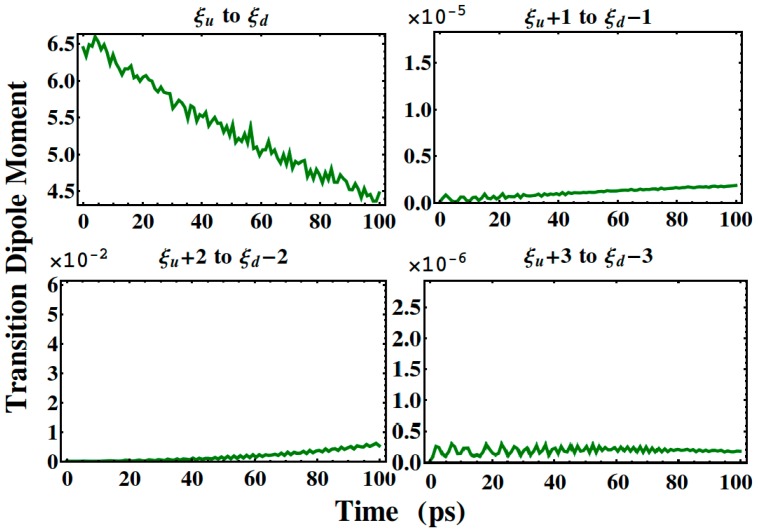
Time evolution of the square of selected sub-dipole moments between different eigenstates (the units of the vertical axis are e^2^Å^2^).

Figure 5 exhibits the evolution of the dipole moment, where it is seen that the transition dipole from eigenstate $|\xi_{u}\rangle$ to $|\xi_{d}\rangle$ is larger than the others by at least three orders of magnitude. Considering the fact that the total transition rate is proportional to the total dipole moment, the major contribution to the total transition comes just from the dipole moment between the localized states $|\xi_{u}\rangle$ and $|\xi_{d}\rangle$. As shown in Figure 4a and Figure 5, in the first 10 ps, the slow changing trend of this dipole moment from state $|\xi_{u}\rangle$ to $|\xi_{d}\rangle$ also determines the behavior of the fluorescence intensity of wavelength 594 nm, where the fluctuating feature reflects the nonadiabatic nature of the real system. Contrary to this, the other transition dipole moments in Figure 5—contributed by the transition from $|\xi_{u}+1\rangle$ to $|\xi_{d}-1\rangle$ and from $|\xi_{u}+2\rangle$ to $|\xi_{d}-2\rangle$—slowly increase in magnitude as time evolves. This raises the question as to how the electron transition influences the exciton delocalization.

**Figure 6:**
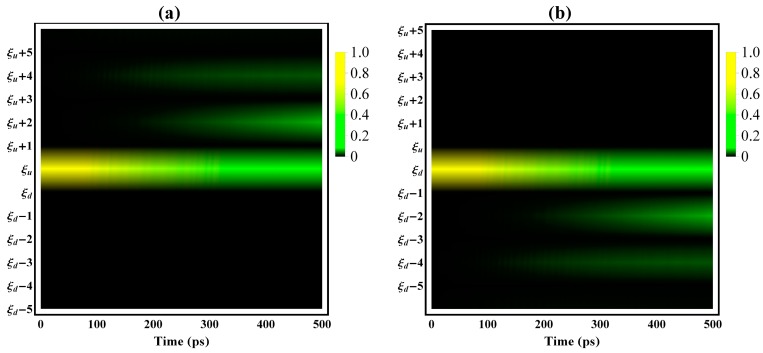
Time evolution of the probability distribution of the exciton over (**a**) molecular orbitals above the gap and the localized orbital $|\xi_{u}\rangle$; and (**b**) molecular orbitals below the gap and the localized molecular orbital $|\xi_{d}\rangle$.

Utilizing brightness to depict the electron populations in the related molecular orbitals, Figure 6a depicts the time-dependent electron population of the exciton distributed over the orbitals above the gap and localized molecular orbital $|\xi_{u}\rangle$, while Figure 6b illustrates the hole of the exciton over the orbitals below the gap and localized molecular orbital $|\xi_{d}\rangle$. At the beginning (as shown in Figure 6), the brightness is almost totally concentrated on the transition between the localized orbitals—i.e., $|\xi_{u}\rangle$ or $|\xi_{d}\rangle$—with the initial state of the radiative decay mostly due to the localized molecular orbital. However, as the decay continues, the brightness in the localized molecular orbitals $|\xi_{u}\rangle$ and $|\xi_{d}\rangle$ becomes dim, while the brightness in the nearby nonlocalized molecular orbitals, such as $|\xi_{u}+2\rangle$ and $|\xi_{d}-2\rangle$, becomes stronger. Thus, the population distribution of the exciton and electron, along with the decay, gradually transfers to the nearby extensive orbitals from the original localized ones, while the extended/delocalized molecular orbitals gradually gain more electron population, leading to the delocalization of the exciton. The delocalization is also affected by the decrease of the non-major transition dipole moments and the increase of the major transition dipole moment in Figure 5. Although the whole timescale of the singlet exciton radiative decay lasts for several hundred picoseconds, Figure 6 indicates that this intrinsic delocalization channel has become rather prevalent, from tens to one hundred picoseconds.

**Figure 7:**
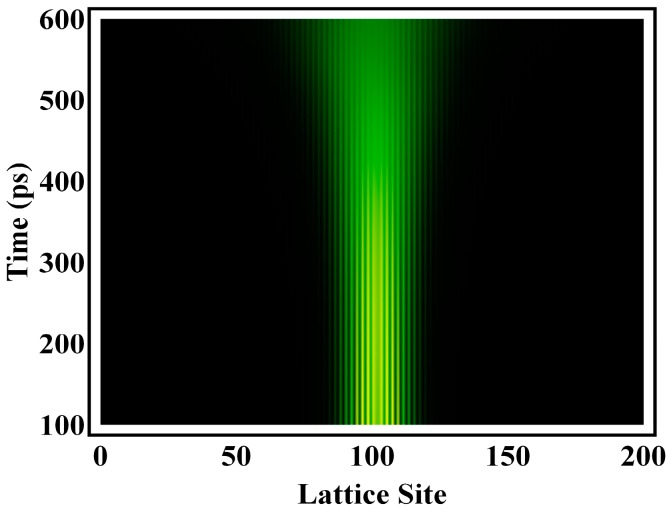
Time evolution of the electronic wavefunction of the exciton.

The physical picture for this delocalization channel is schematically demonstrated in Figure 7. Initially, the electron population largely lies in both localized molecular orbitals in the middle of the gap, which mostly contributes to the localization of the exciton and electron–hole pair. Yet, after about 100 ps, the dipole moment drives the electron to partially transfer to several extended orbitals from the original localized ones, leading to self-delocalization. This figure also depicts the dynamical evolution of the electron of the exciton within 600 ps, where not only does the exciton delocalization take place, but the locally-distorted alternating bonds of the conjugated polymer are also relaxed.

## 4. Conclusions

We have combined the electron–phonon interaction with dynamic transition fluorescence spectra and embedded them into a nonadiabatic mechanism, thus developing unconventional molecular dynamics specifically for the study of the fluorescent decay process of a singlet exciton in a conjugated polymer. Based on the time-dependent fluorescence and evolution of the dipole moment, this article shows that after the exciton is created due to photoexcitation in the conjugated polymer, the dipole moment drives the electron to partially transfer to several extended orbitals from the original localized ones, leading to self-delocalization. The intrinsic delocalization channel therefore acts as a bridge for exciton diffusion in solar cells. This indicates that modification of the dipole moment can enhance the efficiency of polymer solar cells, which is supported by recent experiments [31].
